# Leveraging structured EMR data for efficient patient prescreening: a practical approach to reducing screen-failure rates in Light Touch Trial

**DOI:** 10.1038/s41433-026-04267-w

**Published:** 2026-02-04

**Authors:** Eleonora Riotto, Francesca Lamanna, Adnan H. Khan, Sridevi Thottarath, Hagar Khalid, Swati Chandak, Jessica Bennett, Sarah Hill, Livia Faes, Dun Jack Fu

**Affiliations:** 1 NIHR Biomedical Research Centre at Moorfields Eye Hospital NHS Foundation Trust, UCL Institute of Ophthalmology, London, UK; 2 Cantonal Hospital Winterthur, Winterthur, Switzerland; 3 University of Zurich Epidemiology, Biostatistics & Prevention Institute (EBPI), Zurich, Switzerland

**Keywords:** Medical research, Outcomes research

Screening for clinical trials represents a significant challenge in ophthalmic research due to inefficiencies and costs related to manual review of medical records and ocular imaging [1–4]. Screen failures rates are also high. This constraint is particularly acute in competitive therapeutic areas like neovascular age-related macular degeneration (nAMD), where the identification of eligible patients from a large population of patients undergoing treatment requires meticulous extraction of a highly specific patient subgroup [5–7]. Although manual prescreening is a common practice, the implementation of systematic, automated prescreening using electronic medical record (EMR) data is an emerging and underutilized methodology in ophthalmology trials [8]. Penberthy et al. estimated screening costs to be up to $336.48 per enrolled patient, but this figure considered only personnel expenses for research nurses and clinical associates [9]. For a modern nAMD trial, however, where screening includes advanced imaging and multiple complex assessments, the costs are substantially higher. For instance, within the Light Touch study—a multicentre randomized controlled phase III non-inferiority clinical trial comparing the effectiveness of a lighter versus standard initial dosing regimen of faricimab in pretreated nAMD patients—a screening visit comprising visual acuity checks with refraction, spectral-domain optical coherence tomography (SD-OCT), OCT-angiography (OCTA), and a suite of patient-reported outcomes carries an estimated cost of £2,500 ($3,125 USD) per screened patient. In this commentary, we detail the integration and outcomes of a pragmatic, two-step prescreening strategy for the Light Touch Trial [10]. Our experience offers a replicable model for substantially improving trial efficiency.

The foundation of our approach was the scale of the Moorfields Eye Hospital NHS Foundation Trust (MEH), a network of over 20 sites that treats just under 1000 new nAMD cases annually [11]. This clinical database, encompassing 102,912 intravitreal injections represent a mammoth, untapped asset with a potential pool of 13,576 patients (16,526 eyes) with nAMD from which to recruit. Manually reviewing every chart from this vast cohort would be prohibitively time-consuming. Instead, we designed a two-step funnel to efficiently distill this population. The first step involved an automated EMR-driven prescreening. We programmed searches to identify patients based on a core set of critical, electronically discernible inclusion criteria: a diagnosis of nAMD, actively receiving intravitreal injections, not yet switched to faricimab, an injection interval approximately aligning with the window required for the trial, meeting visual acuity criteria, and absence of recent intraocular surgery. By automating these fundamental eligibility checks, we condensed the initial pool of over 13,000 patients down to a manageable 2365 patient-eye entries for the next stage of review. This refined list then underwent a crucial second step: a confirmatory manual review by trained clinical research staff. This phase aimed to identify exclusion criteria often buried in unstructured clinical notes, such as specific comorbidities or nuanced clinical judgments. Of the 2365 prescreened patient-eye entries, 1718 (73%) were excluded during the confirmatory manual review stage. Primary reasons for exclusion were injection intervals exceeding 12-week limit required for the study and the presence of significant ocular comorbidities, validating the precision of our prescreening methodology in identifying protocol-specific ineligibility. This two-step process yielded a highly vetted list of 647 distinct, pre-qualified patients to approach. Our approach yielded exceptional results: of the 48 invited patients who attended formal screening at MEH, 100% (*n* = 48) met all eligibility criteria, and 98% (*n* = 47) were successfully recruited into the Light Touch Trial. To reach the overall study recruitment target (*n* = 230) with a 2% screening-failure rate, we would need about 235 prescreen-passed candidates. At a 27% prescreen pass rate, only 870 automatically prescreened records would have needed to be manually validated (contra the 2365 carried out!)

**Fig. 1:**
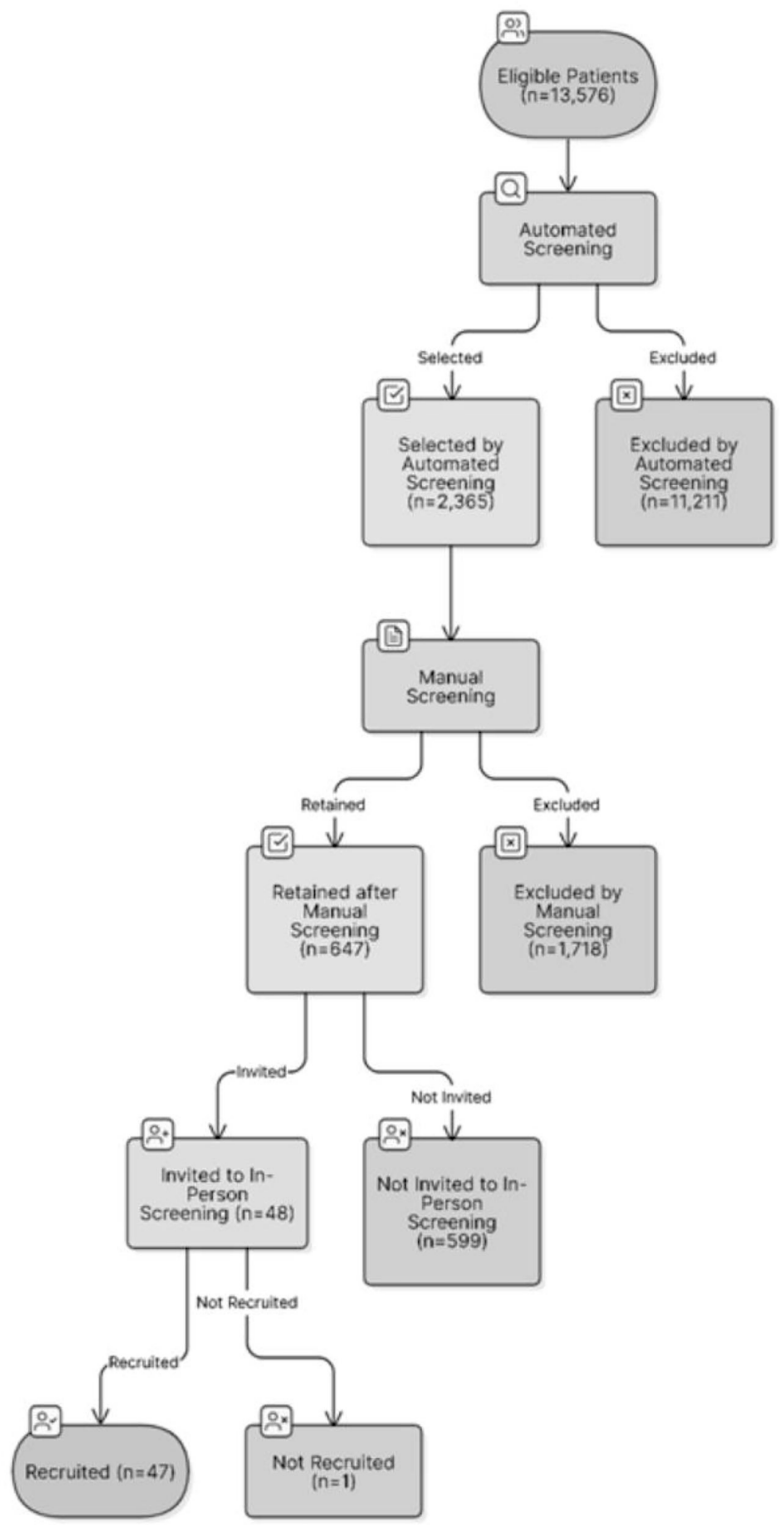
Participant flow through screening and recruitment stages. Presents the CONSORT flow diagram, which outlines the number of patients assessed for eligibility at each step of the prescreening, screening, and recruitment process.

Table 1 summarizes the participant eligibility criteria applied at each stage of the screening process. Figure 1 depicts the flow diagram, showing patient numbers at each stage of prescreening, screening, and recruitment.

**Table 1:** Participant screening stages and applied criteria.

Screening Stage	Formal Criteria Application	Clinician’s Expert Judgment (Manual Screening)
1. Automated Screening	• Aged 50 years or older • Confirmed diagnosis of nAMD in at least one eye • Previously treated with at least three monthly intravitreal anti-VEGF injections (with drugs different from faricimab) • Injection interval approximately aligning with the trial’s window • Meeting broad visual acuity criteria • Absence of recent contraindicating surgery	*(Not applicable)*
2. Manual Screening	• Demonstrates an ongoing need for treatment (i.e., incomplete or unstable disease control)	• Assessed sufficient media clarity and fixation: By scrutinizing recent OCT, fundus photos, and clinical notes for artifacts or pathologies that would preclude quality imaging. • Assessed Likely BCVA ≥ 24 letters: By reviewing recent visual acuity records from clinical notes. • Assessed ability and willingness for follow-up: By contacting the patient directly via telephone to confirm their commitment, logistical feasibility, and understanding of the study requirements.
3. In-Person Screening	• Sufficient media clarity and fixation to permit high-quality imaging • BCVA of at least 24 ETDRS letters • Able and willing to attend regular follow-up visits for the 56-week study period	*(Formal confirmation and final data collection at the visit)*

This table outlines the three-stage screening process, listing the formal eligibility criteria and the corresponding clinician-led assessments applied at each stage.

The automated pre-screening step could evaluate 55% of the trial’s selection criteria. The remaining criteria, which were inherently dependent on manual review by clinicians applying expert judgment. For instance, by scrutinizing recent clinical notes and existing imaging; patients with documented issues like significant cataract or a history of poor compliance were proactively excluded, thereby pre-emptively mitigating a significant portion of the potential screen-failure risk at the formal visit.

The efficacy of the manual pre-screening steps is unequivocally demonstrated by the subsequent screening and enrollment outcomes. Patients from the vetted list were systematically approached to align with their next scheduled clinical appointment. This strategy was beneficial in several key ways: it prevented selection bias by not prioritizing patients who might be more readily available, it safeguarded against patients becoming ineligible if they were randomly contacted later and had already switched treatments, and it was timely for the patient, integrating the trial discussion with their imminent care plan rather than sending preliminary information months in advance.

This efficient cascade from screening to randomization stands in stark contrast to the traditional method of screening a broader, less-vetted population, where screen-failure rates are typically far higher [12–14]. By front-loading the work with intelligent data extraction, we minimized the number of patients who underwent a full screening visit only to fail, thereby conserving valuable clinician time, reducing patient burden, and controlling trial costs.

In conclusion, our EMR-driven prescreening protocol successfully transformed an overwhelming pool of over 13,000 patients into a targeted group of 647 pre-qualified candidates, culminating in a 98% recruitment rate from those screened. This pragmatic, two-step model demonstrates that structured EMR data is a powerful tool for moving clinical trial sites from a reactive to a proactive posture. The methodology is scalable and exportable, representing a tangible step towards smarter, more efficient, and less costly clinical research. As trial designs grow more complex, the wider adoption of such data-driven prescreening methodologies will be essential for the future success of ophthalmic research.
